# Role of immunohistochemistry in lymphoma

**DOI:** 10.4103/0971-5851.76201

**Published:** 2010

**Authors:** I. Satish Rao

**Affiliations:** *Department of Pathology, Krishna Institute of Medical Sciences, Hyderabad, India*

## INTRODUCTION

World Health Organization broadly classifies lymphomas into Hodgkin lymphoma (HL) and non-Hodgkin lymphoma (NHL). Non-Hodgkin lymphoma is further subclassified based on the stage of maturation (immature vs. mature) and cell of origin [B cell, T cell, or natural killer cell (NK) cell].

Morphologic assessment takes into account the anatomic architectural alterations in the lymphoid compartment [i.e., B-cell follicle (follicle center, mantle, or marginal zone) or T-cell regions (interfollicular or sinus areas)]. If an abnormal population is present (polymorphic or monomorphic), the determination of pattern (diffuse or nodular) and cell size (small, intermediate, large) and nuclear characteristics (round, irregular, cleaved with condensed or dispersed or blastic chromatin, and the character of the nucleoli) is made.

The unique feature of lymphomas is the fact that these are considered as clonal proliferation of lymphocytes arrested at different stages of differentiation, thereby recapitulating stages of normal lymphocyte differentiation. Immunohistochemistry (IHC) with various antibodies identifies the specific lineage and developmental stage of the lymphoma.

A panel of markers is decided based on morphologic differential diagnosis (no single marker is specific) which includes leukocyte common antigen (LCA), B-cell markers (CD20 and CD79a), T-cell markers (CD3 and CD5) and other markers like CD23, bcl-2, CD10, cyclinD1, CD15, CD30, ALK-1, CD138 (based on cytoarchitectural pattern).

This review addresses the three-pronged role of IHC in the lymphoma – subtyping, prognostication and potential for targeted therapy in commonly encountered nodal lymphomas. A complete knowledge of the type of positivity (membrane, cytoplasmic nuclear) with awareness of associated caveats is essential for the accurate subtyping and distinguishing from reactive processes.

## ROLE IN SUBTYPING (MORPHOLOGY WITH IHC)[1–5]

The role is given in Tables 1–5

**Table 1:** Polymorphous population

	Basic markers					New/additional markers		
	CD15	CD30	CD20	CD45	ALK−1	Clusterin	MUM1	Fascin
CHD	+	+	−/+	−/+	−	+/− (M)	+	+ Strong (C)
NLPHD	−	−	+	+	−	−	−/+ or −	−
ALCL	−	+	−	+	+	+dot like (C)	+	+ Weak (C)
T/HRBCL	−	−/+	+	+	−	−/+	UK	UK
MLBCL	−	−/+	+	+	−	UK	UK	−

+, >50%; −, <5%; −/+, 5–25%; +/−, 25–50%; (M) – membrane; (C) − cytoplasmic; UK – unknown; CHD - Classical Hodgkin’s disease; NLPHD − Nodular lymphocyte predominance Hodgkin–s disease; ALCL - Anaplastic large cell lymphoma; T/HRBCL - T–cell/histiocyte rich B-cell lymphoma; MLBCL - Mediastinal large B-cell lymphoma; Important caveats to remember: CD15 – 30% of CHD can be negative; CD30 – Can be expressed in embryonal carcinoma melanoma and pancreatic cancer; ALK-1- Variable staining based on the type of chromosomal translocation

**Table 2:** Monomorphic small cell (B-cell neoplasms commonly encountered)

	Basic markers					New/additional markers		
	CD5	CD23	CyclinD1	Bcl2	CD10	CD43	BCL-6	PAX-5
FL	−	−/+	−	+ (Nodules)	+	−	+	−/+
CLL/SLL	+	+	−	+	−	+	−	+
MCL	+	−/+	+	+	−	+	−	+
MZL	−	−	−	+	−	+/−	−/+	+/−

+, >50%, –, >5%; −/+, 5–25%; +/−, 25–50%; FL – Follicular lymphoma; CLL/SLL – Chronic lymphocytic leukemia/small lymphocyte lymphoma; MCL – Mantle cell lymphoma; MZL – Marginal zone lymphoma

**Table 3:** Monomorphic intermediate sized cells with diffuse pattern

	CD20	CD10	Tdt	CD99	Mib-1
Lymphoblastic	−/+ (Btype)	+	+	+	60–70%
Burkitts	+	+	−	−	100%

+, >50%; –, >5%; −/+, 5–25%, +/−, 25–50%;

**Table 4:** Monomorphic large cells with diffuse pattern

	CD20	CD79a	CD138	EBER
DLBCL	+	+	/+	−
Plasmablastic	−	−	+	+

+, >50%; –, >than 5%; −/+, 5–25%; +/− 25–50%; Caveat: CD20 may be negative in Rituximab treated DLBCL; CD79a positivity is confirmatory

**Table 5:** Blastic morphology

	Basic markers					New/additional markers		
	CD20	CD5	CyclinD1	CD23	Tdt	CD10	Pax-5	CD2	CD34
B-ALL/LBL	−/+	−	−	−	+	+	+	−	+
T-ALL/LBL	−	+	−	−	+	+	−	+	+
MCL blastoid variant	+	+	+	−	−	−	+	−	−
FL blastic morphology	+	−	−	+	−	+	+	−	−

+, >50%; –, >5%; −/+, 5–25%; +/− 25–50%; ALL/LBL – Acute lymphocytic leukemia/lymphoblastic lymphoma; MCL – Mantle cell lymphoma; FL – Follicular lymphoma; Caveat: AML and CD56 hematodermic neoplasm are other two tumors in the differential diagnosis

## ROLE IN PROGNOSTICATION

1. CD10, BCL6, and MUM1 expression differentiates two distinct prognostic groups of primary nodal DLBCL - Germinal center type longer survival activated type.[1]
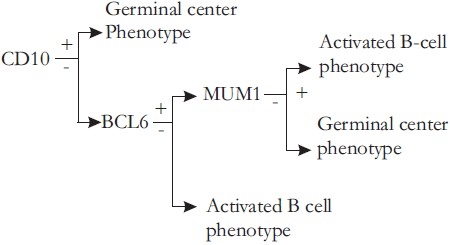
2. BCL-2 positivity with MYC translocations in follicular lymphoma - Aggressive course^2^
3. ALK positive ALCL better prognosis than ALK negative ALCL(5 yr survival 80%vs 48%)^2^

## ROLE IN TARGETED THERAPY[4]

1. Rituximab in B-cell lymphomas has a well established role. Murine models being studied in Hodgkin’s lymphoma along with CD25 (IL-2 receptor)
2. CD22 – IgG 1 antibody (Epratuzumab) in relapsed and refractory, indolent and aggressive NHL ongoing studies evaluating use in conjunction with Rituximab in FL, with CHOP in DLBCL)
3. CD30 – Anti CD30 (SGN 30) in Hodgkin’s lymphoma and CD30 positive T-cell lymphomas (ongoing phase 2 studies)
4. CD40 – Anti CD40 (SGN 40) in recurrent B-cell NHL (ongoing phase 2 studies)
5. CD80 – Anti CD80 (Galiximab) in relapsed and refractory FL in conjunction with Rituximab (ongoing phase 2 studies)

In conclusion, judicious use of panel of antibodies in the light of characteristic cytoarchitectural features helps to recognize the characteristic immunophenotype in most of the lymphomas. Successful outcome with Rituximab has created an interest to search for newer monoclonal antibodies as potential therapeutic targets. Better understanding of the pathogenesis of lymphomas has been possible with the help of IHC.
